# Assessing the Performance of a Modified LACE Index (LACE-rt) to Predict Unplanned Readmission After Discharge in a Community Teaching Hospital

**DOI:** 10.2196/ijmr.7183

**Published:** 2017-03-08

**Authors:** Christo El Morr, Liane Ginsburg, Seungree Nam, Susan Woollard

**Affiliations:** ^1^ Faculty of Health School of Health Policy and Management York University Toronto, ON Canada; ^2^ North York General Hospital Medicine North York General Hospital Toronto, ON Canada

**Keywords:** patient readmission, hospital readmissions, health services, quality improvement, quality of health care, cost savings, eHealth, medical informatics

## Abstract

**Background:**

The LACE index was designed to predict early death or unplanned readmission after discharge from hospital to the community. However, implementing the LACE tool in real time in a teaching hospital required practical unavoidable modifications.

**Objective:**

The purpose of this study was to validate the implementation of a modified LACE index (LACE-rt) and test its ability to predict readmission risk using data in a hospital setting.

**Methods:**

Data from the Canadian Institute for Health Information’s Discharge Abstract Database (DAD), the National Ambulatory Care Reporting System (NACRS), and the hospital electronic medical record for one large community hospital in Toronto, Canada, were used in this study. A total of 3855 admissions from September 2013 to July 2014 were analyzed (N=3855) using descriptive statistics, regression analysis, and receiver operating characteristic analysis. Prospectively collected data from DAD and NACRS were linked to inpatient data.

**Results:**

The LACE-rt index was a fair test to predict readmission risk (C statistic=.632). A LACE-rt score of 10 is a good threshold to differentiate between patients with low and high readmission risk; the high-risk patients are 2.648 times more likely to be readmitted than those at low risk. The introduction of LACE-rt had no significant impact on readmission reduction.

**Conclusions:**

The LACE-rt is a fair tool for identifying those at risk of readmission. A collaborative cross-sectoral effort that includes those in charge of providing community-based care is needed to reduce readmission rates. An eHealth solution could play a major role in streamlining this collaboration.

## Introduction

Unplanned hospital readmission has been a major challenge in health care worldwide [1]. In the United States, as of 2012, the Hospital Readmissions Reduction Program has been measuring hospital readmission rates and penalizes hospitals with excessive readmission rates [2]. In Canada, 8.5% of patients are readmitted within a month of their discharge [3]. Medical patients have the highest rate of readmission (13%), followed by surgical and pediatric patients (6.5%). The financial consequence of readmission is estimated at Can $1.8 billion [4]. Recent studies suggest that 9%-59% of unplanned readmissions are preventable when appropriate measures are instituted [5-7]. Postdischarge interventions are effective [8]; however, they are resource intensive and costly. Identifying patients associated with higher risk of readmission may be a more cost-effective way to reduce readmissions. Rather than focusing on readmission risk factors for specific medical conditions as others have done [9,10], van Walraven and colleagues [11] developed the “LACE” index, a cross-conditions tool that predicts early death or unplanned readmission after discharge from hospital. The LACE index is composed of data on “ *L* ength of stay” in the hospital during the current admission, “ *A* cuity of admission” (acute or not), “ *C* omorbidity of patient” (measured using the Charlson comorbidity index) [12,13], and “ *E* mergency department use” in the 6-month period before the current admission. In teaching settings, van Walraven et al [11] reported that a 1-point increase in the LACE score increased the odds of unplanned readmission by 18% and the odds of early death by 29%. Other work, also in teaching settings, found that patients identified as high-risk patients using the LACE tool (LACE score≥10) were readmitted twice as often as other patients and had slightly longer lengths of stay [14]. Mixon et al [15] reported that the LACE index is a better predictor of readmission than measures of patient self-reported preparedness for discharge.

Other tools addressing hospital readmission, such as the UK Nuffield Trust model [16] and the Scottish Patients at Risk of Readmission (SPARRA) [17], exist. The UK Nuffield Trust model was developed in the United Kingdom to identify patients at highest risk of emergency admission and is based on 88 variables extracted from complete hospital and general practitioners’ systems. SPARRA is a predictive risk stratification tool developed in Scotland to evaluate a person’s risk of being admitted to hospital as an emergency inpatient within the next year. SPARRA holds promise for (1) jurisdictions where resources are devoted to a preventive approach to patient management across the health system and (2) health systems with linked datasets from general practice, home and community care settings, pharmacies, and other settings that allow risk scores to be calculated for large portions of a population [18]. Many jurisdictions continue to face considerable barriers to this level of system and data integration. In such jurisdictions, focusing on reducing readmission using the LACE-rt index remains viable.

While van Walraven et al developed LACE based on a secondary analysis of a multicenter, prospective cohort study of patients in 11 hospitals, our study examined the use of a modified LACE index (LACE-rt) tailored for use in real time in an active setting in the general medicine unit at a large community teaching hospital in Toronto. In order to use the LACE tool in real time to help identify those discharged patients who are at higher risk of readmission, some practical unavoidable modifications had to be made to the LACE index. Accordingly, the purpose of this study was to implement a modified LACE index in a real-time setting (hence the name LACE-rt) and examine its reliability as well as its ability to discriminate between high- and low-risk patients.

## Methods

### Data Sources and Study Population

The hospital is a community teaching hospital with 426 acute care beds. Secondary data covering the period September 2013 to July 2014 were obtained from the hospital. A total of 3 datasets were provided:

1. Inpatient information extracted from the Canadian Institute for Health Information’s Discharge Abstract Database (DAD); it included patient identifier, encounter identifier, admission and discharge dates, location of admission, and basic demographic information such as age and sex.
2. Emergency department visit data extracted from the National Ambulatory Care Reporting System (NACRS).
3. “LACE-rt” related information extracted from the hospital electronic medical record.

### Inclusion Criteria

The data included 7676 admissions from 6332 patients. Among these admissions, we selected those who were admitted to 1 of the 4 medicine units that implemented LACE-rt (Stroke, Acute Geriatrics, Cardiology, and Respirology and Gastrointestinal) and were assessed by a nurse using the LACE-rt tool before being discharged to home, another hospital, or a long-term care facility. The total number of admissions analyzed in our study was 3855 (N=3855).

### The LACE-rt Score

The “L” value is calculated differently in LACE-rt than in the original LACE index. When managers at the hospital decided to implement the LACE index, they faced the practical challenge of needing to start preparing for discharge as soon as the patient is admitted; waiting until the discharge day to compute the “L” score would delay discharge planning, making the original LACE approach untenable from a practical standpoint. In LACE-rt the managers therefore decided to compute “L” based on the patient’s length of stay during the previous (instead of current) acute care admission within the last 30 days.

The attributes L, A, C, and E are computed in the same way in LACE-rt and the original LACE; their corresponding values and points are provided in Table 1. For attribute L, the value column displays the length of stay in days, during the previous admission (LACE-rt) or the current one (original LACE). For attribute A, the value column displays *yes* for acute admissions, *no* otherwise. For attribute C, the value column displays the Charlson comorbidity index score. For attribute E, the value column displays the number of visits to the emergency department within the last 6 months. To each attribute’s value correspond a number of points. The sum of all points assigned to L, A, C, and E constitutes a LACE index (LACE-rt or original LACE).

The Charlson comorbidity score (C) is calculated as follows: 1 point for history of myocardial infarction, peripheral vascular disease, cerebrovascular disease, or diabetes without complications; 2 points for congestive heart failure, chronic obstructive pulmonary disease, mild liver disease or cancer, diabetes with end-organ damage, and any tumor (including lymphoma or leukemia); 3 points for dementia or connective tissue disease; 4 points for moderate to severe liver disease or human immunodeficiency virus infection; and 6 points for metastatic cancer.

Both the original LACE index and the LACE-rt index scores range from 0 to 19, where a higher score indicates an increased chance of readmission or early death (Table 1).

**Table 1:** The LACE and LACE-rt index attributes and the corresponding values and points.

Attribute	Value	Points
Length of stay^a^, days	<1	0
	1	1
	2	2
	3	3
	4-6	4
	7-13	5
	≥14	7
Acute (emergent) admission	Yes	3
	No	0
Comorbidity (Charlson comorbidity index score)	0	0
	1	1
	2	2
	3	3
	≥4	5
Emergency department visit (within the last 6 months)	0	0
	1	1
	2	2
	3	3
	≥4	4

^a^LACE: during the current admission (van Walraven et al); LACE-rt: during the last 30 days.

In this study, nurses checked the hospital’s electronic patient chart to estimate the values for “L,” “A,” “C,” and “E,” then entered those values into a software interface that computes the patient’s LACE-rt score. However, discussion with staff suggested that the extraction and recording of the “L,” “E,” and “C” values are often done quickly.

### Calculations

To check the data entry accuracy for the “L” and “E” components in our dataset, we computed “L” and “E” using the DAD and NACRS data, respectively, and compared the calculations from the administrative data with those values entered manually by the nurses. Even though we had a rationale for investigating the accuracy of “C,” this was not feasible as it would have required a complex time-consuming clinical assessment.

### Outcome Variables

**Figure 1:**
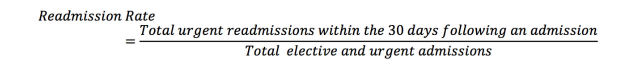
Readmission rate formula.

According to Statistics Canada, “non-elective return to an acute care hospital for any cause is counted as a readmission if it occurs within 30 days of the index episode of inpatient care” [19]. Similarly, we have defined an “unplanned hospital readmission” as an urgent rehospitalization of the patient within 30 days of discharge, excluding patient’s elective readmission to the hospital. Thus, the formula for calculating the readmission rate is computed as shown in Figure 1.

### Statistical Analysis

Statistical analyses were performed using IBM SPSS Statistics 22 (IBM Corporation). Descriptive statistical analysis was carried out describing the population’s basic demographic characteristics. On the basis of previous literature, patients with LACE-rt score of 10 or higher were defined as a high-risk group and those with a score lower than 10 were defined as the low-risk group [14]. The readmission rates of these 2 groups were then compared using chi-square analysis. To further support the chi-square analysis and to measure the difference between the low- and high-risk groups, a binary logistic regression analysis was carried out to compare the odds ratio of LACE-rt scores ≥10 and LACE-rt scores <10 in relation to readmission. The odds ratio gave the magnitude of the difference between low- and high-risk groups. Accuracy of the LACE-rt score in predicting readmission was assessed using receiver operating characteristic (ROC) analysis and the C statistic. The C statistic measures the discriminatory power of a prediction model [20]; it reflects the probability that the measure (in this case the LACE-rt index) is higher for a case (ie, a readmission) than for a noncase [21].

This project obtained ethical approval from the hospital Research Ethics Board and all researchers obtained the “Tri-Council Policy Statement: Ethical Conduct for Research Involving Humans Course on Research Ethics” certificate (TCPS 2: CORE).

## Results

### Descriptive Statistics

Descriptive statistics (Table 2) showed that during the period of study (September 2013 and July 2014), 51.78% of hospital admissions were female patients. During the same period, most patients admitted to hospital were elderly. Almost half of the admitted patients were 80 years of age or older, and more than 80% of the patients were 60 years of age or older. The mean age was 74.29 years.

**Table 2:** Descriptive statistics: patients’ sex and age groups.

Hospital admission characteristics (N=3855)	Value	Count (%)
Sex	Male	1859 (48.22)
	Female	1996 (51.78)
Age, years	Mean age	74.29
	<20	10 (0.26)
	20-29	82 (2.13)
	30-39	99 (2.57)
	40-49	182 (4.72)
	50-59	346 (8.98)
	60-69	484 (12.56)
	70-79	763 (19.79)
	≥80	1889 (49.00)

Table 3 describes our sample for each of the LACE-rt elements. A total of 94% of patients were admitted for less than 1 week and 5.9% remained in hospital for more than 1 week; however, the majority (2559/3855, 66.38%) stayed for less than 1 day. Of the admissions, 95.77% were not acute. On the Charlson comorbidity index, 30.06% of patients scored zero, 25.40% scored 1, and almost 45% scored 2 or more. A total of 27.34% of patients were seen in the emergency department at least twice in the 6-month period before the index admission.

**Table 3:** Descriptive statistics: LACE-rt elements and their corresponding frequencies.

LACE-rt elements (N=3855)	Value	Count (%)
Length of stay in the last 30 days	Less than 1 day	2559 (66.38)
	1 day	648 (16.81)	
	2 days	148 (3.84)
	3 days	94 (2.44)
	4-6 days	179 (4.64)
	7-13 days	134 (3.48)
	≥14 days	93 (2.41)
Acute (emergent) admission	Yes	163 (4.23)
	No	3692 (95.77)
Comorbidity (Charlson comorbidity index score)	0	1159 (30.06)
	1	979 (25.40)
	2	625 (16.21)	
	3	559 (14.50)
	≥4	533 (13.83)
Emergency department visit (within the last 6 months)	0 visits	1776 (46.07)
	1 visit	1025 (26.59)
	2 visits	541 (14.03)
	3 visits	246 (6.38)
	≥4 visits	267 (6.93)

### Readmission Rates

Differences between the high- and low-risk groups were compared in a cross-tabulation. The readmission rate for the low-risk group was 10.6% compared with 23.9% for the high-risk group. The chi-square analysis indicated that there is a statistically significant difference between the 2 groups (χ^2^_1_=65.5, N=3855, *P*<.001).

**Figure 2:**
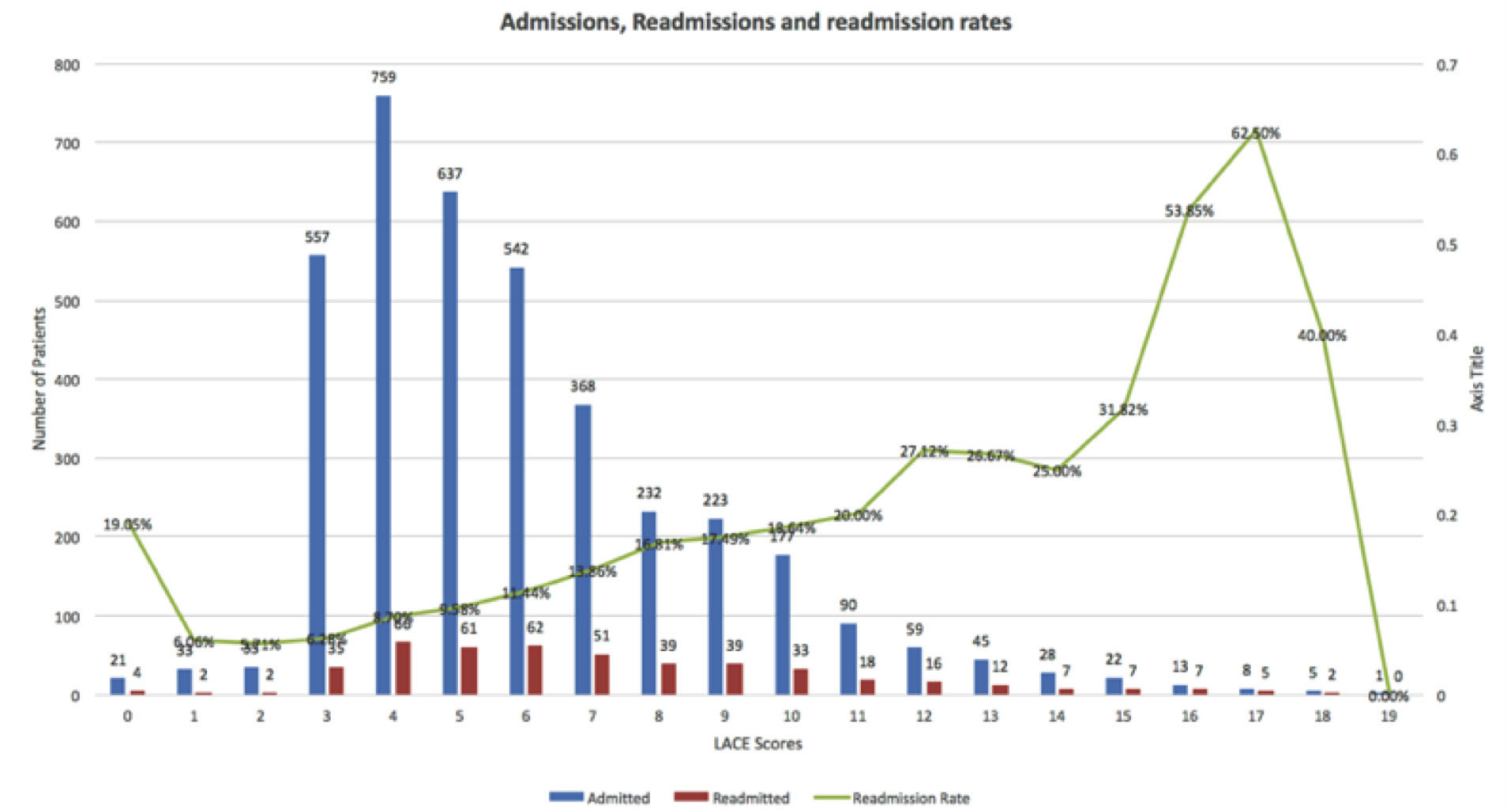
Readmission rates by LACE-rt scores.

Figure 2 shows readmission rates for the range of LACE-rt scores. There is a sharp decrease at LACE-rt scores 18 and 19; however, there are a very small number of patients for these 2 scores (7 and 1, respectively).

### Predictive Power of LACE-rt at the Hospital: High-Risk Versus Low-Risk Patients

The binary logistic regression analysis revealed an odds ratio Exp(B)=2.648, *P*<.001, which indicated that the patients in the high-risk group are 2.65 times more likely to be readmitted than those in the low-risk group. Data revealed that some patients with a low LACE-rt score were being readmitted. We examined whether reducing the LACE-rt threshold from 10 to 8 would have better predictive power by allowing us to capture more of the high-risk patients. The logistic regression results showed that LACE-rt would have less predictive power with a threshold of 8 (odds ratio Exp(B)=2.43).

### Readmission Rates by Age Groups and Sex

Of the readmissions, 11.9% were for female patients and 12.4% were for male patients. An analysis of the readmission rates by sex indicated that there is no significant difference between the 2 groups (χ^2^_1_=.3, *P*=.60).

An analysis of the readmission rates by age groups indicated that the readmission rates were 10%, 7.3%, 5.1%, 11.0%, 7.2%, 11.2%, 11.4%, and 14.3% for the age groups <20 years, 20s, 30s, 40s, 50s, 60s, 70, and ≥80 years, respectively (χ^2^=23.6, *P*<.001). However, looking at readmitted patients alone, 57.7% of them were of age 80 years or older, 18.6% were in their 70s, and 11.5% were in their 60s—in total, close to 90% of readmitted patients were aged 60 years or older.

### Readmissions Before and After the LACE-rt Implementation

We used the nonparametric Mann-Whitney *U* test to assess whether the introduction of the LACE-rt tool had any impact on readmission rates. There was no significant difference in readmission rates between the period before the LACE-rt and after the LACE-rt implementation (*U*=126,000, *P*=.23).

### Receiver Operating Characteristic Analysis

To assess the accuracy of the LACE-rt index in predicting readmission, we conducted an ROC curve analysis. The ROC analysis was statistically significant (*P*<.001). The C statistic for the LACE-rt index as a predictor of readmission was .632 (95% CI 0.604-0.659). A C statistic value between .8 and .89 indicates an excellent test, a value between .7 and .79 indicates a good test, and a value between .51 and .69 indicates a poor test [20]. In previous studies, C statistic values of .6 [22] and .65 [23] were reported as indicating a fair test; consequently, it is safe to state that in our hospital environment LACE index was found to be a fair test in predicting readmission.

## Discussion

### Principal Findings

Our results suggest that the LACE-rt index can predict readmission with a reasonable degree of accuracy and that a threshold of 10 is useful for differentiating between patients who are at high versus low risk of readmission. Our results further showed that the readmission rates at the hospital are 10.6% and 23.9% for the low-risk and high-risk groups, respectively. These results are consistent with Gruneir and colleagues [14] who found readmission rates of 9% and 19% for low-risk and high-risk patients, respectively, using the same LACE cutoff.

Current discussion of readmissions in the literature often focuses on demographic and socioeconomic status (SES) factors that explain readmission in specified populations (eg, patients with congestive heart failure). However, demographic and SES predictors are not routinely collected by hospitals; moreover, hospitals would benefit more from tools that work across multiple conditions rather than tools that are specific to certain health conditions. Van Walraven and colleagues recently improved the predictive power of LACE by incorporating age and sex into LACE+ [24]. We suggest that hospitals might collect additional demographic and SES data at the time of admission to better understand which factors are most highly associated with readmission. Such an approach would allow hospitals to use a modified LACE tool, in real time, to identify discharged patients at higher risk of readmission.

The original LACE index required a modification in order to implement it in a hospital setting. As mentioned above, the “Length of stay” could not be implemented in the manner originally designed and had to be modified to measure patients’ length of stay in the last 30 days instead of during the current admission. However, our results suggest that the LACE-rt index remains useful for identifying patients at high risk of readmission. In our sample, higher LACE-rt scores were associated with higher readmission rates. Moreover, the chi-square analysis indicated that patients with a LACE-rt score of ≥10 were significantly more likely to be readmitted than those with a LACE-rt score of <10. This is particularly interesting given no demographic or SES factors were used in these analyses—although most admitted patients we studied were elderly, the LACE-rt tool was still able to distinguish between the high- and low-risk groups.

The ROC analysis showed a C statistic that is lower than the one found in the population studied by van Walraven et al (C statistic .684, 95% CI 0.679-0.691) [11]. The lower C statistic value means that the LACE-rt index had poorer performance in our hospital population than in the population studied by van Walraven et al. This difference in performance is expected, as the characteristics of the 2 populations differed; our population had a mean age of 74.29 years compared with 61.3 years in the population studied by van Walraven et al and LACE index is known to perform poorer in older populations [22,23,25].

Our analysis showed that the LACE-rt implementation itself had no effect on readmission rates. Although hospitals can use the LACE-rt tool to identify patients at high risk of readmission, it is unlikely that use of this type of tool *alone* will reduce readmission rates. Reducing readmission requires intervention and it is an endeavor that likely needs to extend beyond the hospital setting to include coordination with other stakeholders such as family caregivers and other sectors including primary care and agencies responsible for providing home- and community-based care [26]. Processes that may promote such coordination include health informatics solutions that can support the coordination process, including communication among the stakeholders as well as follow-up care and monitoring. Addressing avoidable readmissions will also require policies that support a collective cross-sectoral effort, such as sufficient budgeting for community- and home-based health services, availability of long-term care beds, and eHealth solutions. eHealth solutions such as Web-based communities [27-31] or telemonitoring applications [32-34] for patients with chronic diseases currently being tested to keep patients healthy at home may be helpful for curbing readmission rates.

### Limitations

Our study was not able to take early death into account. Patients who died would appear as patients with no readmission in our dataset. It is therefore likely that our data underestimate actual readmission rates. The fact that we were only able to examine readmissions to the same hospital further contributes to underestimation of our readmission rates.

These limitations should not detract from the purpose of this study, which was to examine utility of the LACE-rt index as a tool for quality improvement. Indeed, methodological concerns related to the measurement of readmission rates have led to suggestions that readmission data are better suited to quality improvement than accountability purposes [35].

### Conclusions

Our main research aim was to examine the extent to which the LACE-rt index could be used as a predictor of readmission in real time in a large community hospital setting. Our results suggest the LACE-rt index can be practically applied and is a good predictor of readmission. We suggest exploring ways to incorporate basic demographic and socioeconomic data into the tool. We already know that geography has an impact on patient’s health [36]. Incorporation of simple geographic location data for admitted patients could shed light on the underlying socioeconomic and sociocultural factors that influence readmissions. Finally, collaborative, cross-sectoral approaches that capitalize on innovative eHealth solutions are required so that we can intervene in the system to reduce costly, often avoidable, and potentially harmful readmissions.

## Abbreviations

DAD: Discharge Abstract Database
NACRS: National Ambulatory Care Reporting System
ROC: receiver operating characteristic
SES: socioeconomic status
SPARRA: Scottish Patients at Risk of Readmission
